# Natural language processing and network analysis provide novel insights on policy and scientific discourse around Sustainable Development Goals

**DOI:** 10.1038/s41598-021-01801-6

**Published:** 2021-11-17

**Authors:** Thomas Bryan Smith, Raffaele Vacca, Luca Mantegazza, Ilaria Capua

**Affiliations:** 1 Bureau of Economic and Business Research, University of Florida, Gainesville, USA; 2 Department of Sociology and Criminology and Law, University of Florida, Gainesville, USA; 3 One Health Center of Excellence, IFAS, University of Florida, Gainesville, USA

**Keywords:** Sustainability, Computer science

## Abstract

The United Nations’ (UN) Sustainable Development Goals (SDGs) are heterogeneous and interdependent, comprising 169 targets and 231 indicators of sustainable development in such diverse areas as health, the environment, and human rights. Existing efforts to map relationships among SDGs are either theoretical investigations of sustainability concepts, or empirical analyses of development indicators and policy simulations. We present an alternative approach, which describes and quantifies the complex network of SDG interdependencies by applying computational methods to policy and scientific documents. Methods of Natural Language Processing are used to measure overlaps in international policy discourse around SDGs, as represented by the corpus of all existing UN progress reports about each goal (N = 85 reports). We then examine if SDG interdependencies emerging from UN discourse are reflected in patterns of integration and collaboration in SDG-related science, by analyzing data on all scientific articles addressing relevant SDGs in the past two decades (N = 779,901 articles). Results identify a strong discursive divide between environmental goals and all other SDGs, and unexpected interdependencies between SDGs in different areas. While UN discourse partially aligns with integration patterns in SDG-related science, important differences are also observed between priorities emerging in UN and global scientific discourse. We discuss implications and insights for scientific research and policy on sustainable development after COVID-19.

## Introduction

Since their conception at the Rio + 20 conference, the United Nations’ Sustainable Development Goals (SDGs) were intended to be both independent, each addressing a distinct sustainability domain, and interconnected, reflecting the many potential interdependencies, synergies and trade-offs between the 17 goals (Table 1) and their 169 constituent targets^1^. These interdependencies are diverse, complex, and potentially varying in space and time. Mapping and understanding them is essential to achieve policy coherence in sustainable development^2–4^, harmonizing and integrating policy action in different sustainability sectors to maximize mutual reinforcements and minimize conflicting outcomes^4–8^. The study of interlinkages between SDGs has garnered increasing attention among sustainability scholars in recent years^9^. At the same time, there have been growing calls for a reorganization and prioritization of policy and scientific efforts on sustainable development around a few, coherent sets or clusters of integrated SDGs^3,10,11^, such as the six “entry points” of the most recent UN Global Sustainable Development Report^12^.

**Table 1:** Sustainable development goals: abbreviation and full descriptions.

Abbreviation	Description
1-Poverty	End poverty in all its forms everywhere
2-Hunger	End hunger, achieve food security and improved nutrition and promote sustainable agriculture
3-Health	Ensure healthy lives and promote well-being for all at all ages
4-Education	Ensure inclusive and equitable quality education and promote lifelong learning opportunities for all
5-Gender	Achieve gender equality and empower all women and girls
6-Sanitation	Ensure availability and sustainable management of water and sanitation for all
7-Energy	Ensure access to affordable, reliable, sustainable and modern energy for all
8-Economy	Promote sustained, inclusive and sustainable economic growth, full and productive employment and decent work for all
9-Industry	Build resilient infrastructure, promote inclusive and sustainable industrialization and foster innovation
10-Inequality	Reduce inequality within and among countries
11-Settlements	Make cities and human settlements inclusive, safe, resilient and sustainable
12-Consumption	Ensure sustainable consumption and production patterns
13-Climate	Take urgent action to combat climate change and its impacts
14-Aquatic	Conserve and sustainably use the oceans, seas and marine resources for sustainable development
15-Terrestrial	Protect, restore and promote sustainable use of terrestrial ecosystems, sustainably manage forests, combat desertification, and halt and reverse land degradation and halt biodiversity loss
16-Peace	Promote peaceful and inclusive societies for sustainable development, provide access to justice for all and build effective, accountable and inclusive institutions at all levels
17-Partnerships	Strengthen the means of implementation and revitalize the global partnership for sustainable development

However, the extant scholarship about SDG interactions and clusters has important limitations, typically relying on manual coding of SDG descriptive texts, qualitative reviews of scientific documents and expert opinions, or observed covariance in SDG indicator data. Building on and complementing this literature, the present study proposes novel empirical methods for identifying SDG interconnections by applying Natural Language Processing (NLP) tools and network science techniques to official UN progress reports and scientific publications around SDGs. We seek to address two main questions: (i) What interconnections and clusters of interrelated SDGs emerge from official UN *policy discourse* around sustainable development? (ii) Are similar interconnections also found in *scientific discourse* about the SDGs?

At the intersection of linguistics, computer science, and machine learning, NLP techniques can be used to detect key words and phrases, extract topics and, crucially for this study, quantify overlaps and relationships between texts^13–15^. These relationships can be analyzed as networks, uncovering complex structures of proximity and distance between documents. In the first part of the article, we use this combination of methods to (i) describe and summarize international policy discourse around SDGs, as represented by official UN reports about the progress made yearly towards each goal; (ii) map networks of SDG interconnections that emerge from UN policy discourse; and (iii) identify clusters of SDGs which the UN tends to discuss similarly, implying they could be addressed simultaneously by policymakers or scientists.

The first wave of scholarship about SDG interdependences was dominated by theoretical classifications of SDGs and their relationships. Attempts to identify clusters of SDGs pertaining to similar domains^16,17^ tended to group them by focus on the natural environment (SDGs 6, 12, 13, 14, 15); on basic human needs or well-being (SDGs 1, 2, 3, 4, 5, 10, 16); and on services, prosperity, and economy (SDGs 2, 6, 7, 8, 9, 10, 11, 12). More granular theoretical classification schemes exist, dividing these broader categories into sub-areas, such as sustainable resource use and earth preconditions within the ecological domain^18^. Such models are limited by their exclusive attention to SDG classification and clustering, ignoring pairwise relationships between goals and the complex networks they form.

Networks of SDG interdependencies were the subject of a second wave of research. A seminal study in this area quantified the degree of synergy between SDGs by examining targets that are shared or cross-referenced in their official descriptions, targets, and indicators^6^. However, this effort was criticized for failing to account for trade-offs and areas of potential conflict between goals^5,19^. A well-known framework developed by the International Science Council (ICSU) indexed interlinkages between SDGs on a 7-point scale ranging from “cancelling” to “indivisible”^7,20^. This approach offers a more comprehensive means of describing SDG synergies and trade-offs, and has since been widely used to quantify the strength and direction of the relationships in SDG networks^2,3,21^. This work has mostly relied on qualitative appraisal of literature by experts, or on qualitative coding and evaluation of SDG descriptive texts. However, in the absence of quantitative and reproducible methods to measure SDG interlinkages, it is difficult to know how the relationships described in this body of research are influenced by the professional backgrounds and subjective views of experts and coders, rather than incorporate objective characteristics of policy, scientific discourse, or social and environmental phenomena relevant to the SDGs.

A third, growing wave of scholarship has begun assessing interdependencies between SDGs based on empirical data on the covariance between SDG indicators^4,22–26^. These studies, often based on system dynamics, have used longitudinal indexes of sustainable development progress to identify SDGs on which advances are made concurrently, and those whose indicators trend in opposite directions^23–26^. Efforts are also being made to collect data for the empirical application of the ICSU framework, but this literature is still in its infancy^8,27^. While this scholarship provides crucial insights about SDG interlinkages, it requires costly production and monitoring of goal indicators and is hindered by the scarcity of indicator data for certain SDGs and targets^4,21,22,28^. Further, this type of work focuses on simultaneous covariance in SDG indices, failing to capture lagged interdependencies such as those between certain social and environmental indicators^3^.

Together with policy coherence, scientific integration around SDGs has emerged as a central challenge in recent years. Scientific evidence and knowledge relevant to SDGs are dispersed and siloed across different disciplines, institutions, geographical scales, and locations—a fragmentation that creates a critical impediment to the advancement of science and policy on sustainable development^3,10,29,30^. In particular, natural science research has been criticized for failing to incorporate insights from the social sciences and environmental humanities, resulting in limited ability to translate scientific findings into positive change^31^. In the second part of this article, we turn attention to scientific integration around SDGs and ask if global scientific debate agrees with international policy discourse on areas of SDG interconnection. We address this question by examining the extent to which the strongest SDG interdependencies emerging from UN official documents are reflected in scientific integration within sustainable development research. When topic combination and collaboration in SDG-related science align with the SDG interconnections observed in global policy discourse, we can also conclude that there is stronger evidence in support of the identified interdependencies between SDGs. Recent scientometric scholarship has investigated scientific collaboration and diversity in research teams in terms of interest areas, disciplines, geography and organizational affiliations, and their effects on scientific production^32–35^. We draw on this literature to examine collaboration and diversity in scientific articles classified by SDG relevance.

To the best of our knowledge, this is the first study to apply NLP methods (in combination with network analysis) to the problem of uncovering SDG interconnections^9^. Importantly, unlike prior research based on expert elicitation, we do not rely on our own (or others’) appraisal of the intensity and direction of SDG interactions. Moreover, unlike studies of indicator covariance, our method is not constrained by the scarcity of indicator data or limited to analysis of simultaneous SDG interactions. Supplementing existing approaches and overcoming some of their limitations, the methods presented here provide a new lens for the study of SDG interactions and could become integral to ongoing efforts to map SDG interlinkages and clusters, and advance both policy coherence and scientific integration on sustainable development.

## Materials and methods

We analyzed the corpus of all “Progress and Information” (P/I) reports presented by the UN Economic and Social Council (ECOSOC) to the UN Secretary General about each SDG each year, from 2016 (the first year such reports were produced) to 2020 (the year of the last available reports)^36^. The reports describe annual global progress made toward each SDG and provide other descriptive information about each goal. All 85 reports (17 SDGs, 5 years) were scraped and cleaned with standard text pre-processing procedures and concatenated by SDG. The length of the resulting 17 P/I documents ranged from 1662 to 6743 tokens (mean = 3092, sd = 1363 tokens). To distill salient information, we applied standard NLP text preprocessing steps, including tokenization, lemmatization, and part-of-speech (POS) tagging. To describe the corpus content we applied the TextRank algorithm to the lemmatized contents of the documents^13,37^. Further details about text preprocessing and results of TextRank analysis are presented in the SI Appendix.

**Figure 1:**
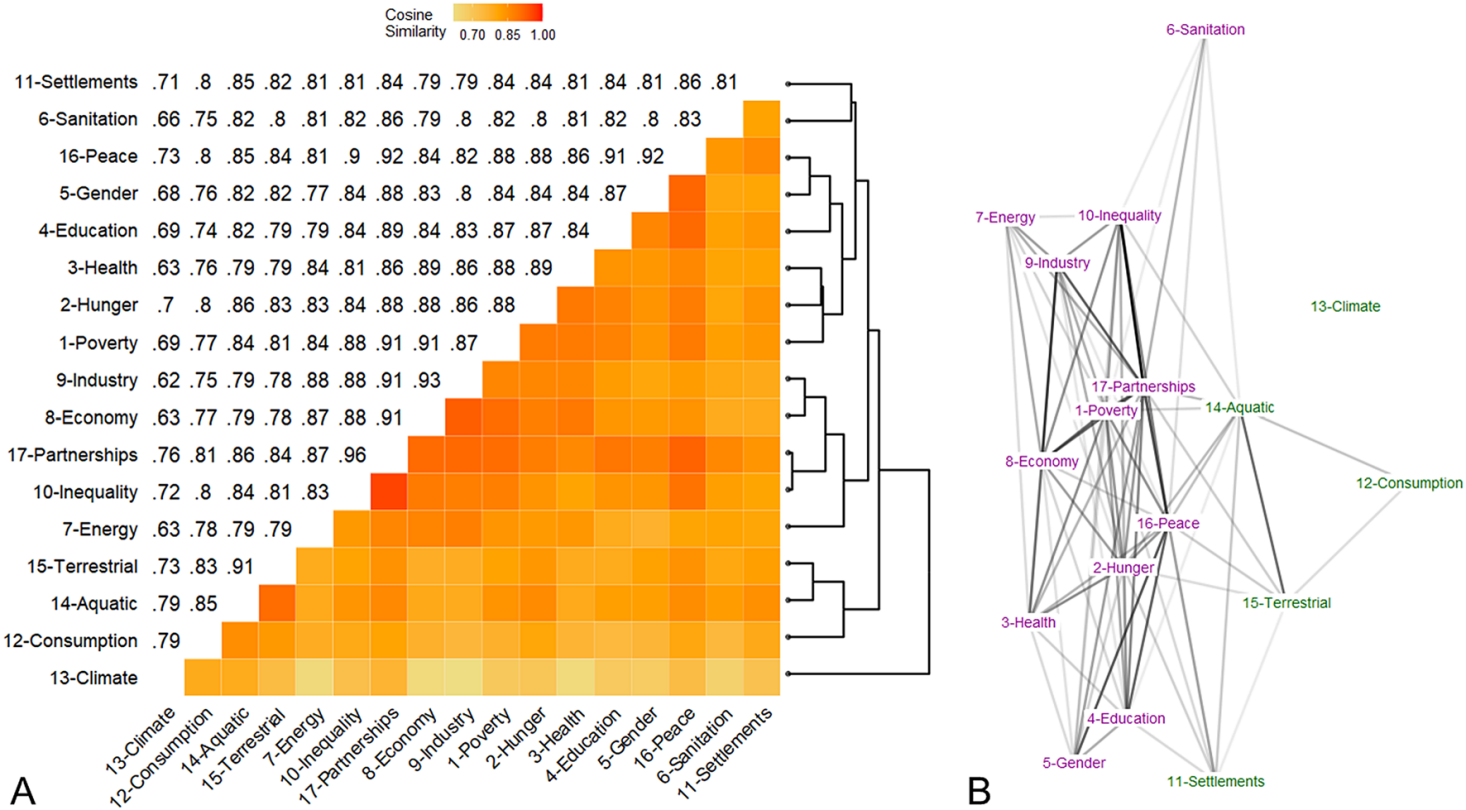
Discursive overlap between SDGs based on cosine similarity in doc2vec embeddings. **(A)** Heatmap of cosine similarity matrix with dendrogram of matrix hierarchical clustering. **(B)** Weighted network of cosine similarities (node colors represent subgroups identified via the Louvain network community detection algorithm^40^).

To map the interdependencies between the SDGs we required distributed numeric representations for which we could calculate similarity measures. This was achieved using a document embedding model (doc2vec), a technique used in NLP to generate numeric representations of documents after inheriting word semantics based on their use in similar lexical contexts in a corpus of training data^15^. A continuous bag-of-words (CBOW) doc2vec model (5-word window) with 300 dimensions and 250 training iterations was then used to quantify semantic overlap between the P/I documents^38^. The doc2vec model introduces a document ID parameter into a CBOW word2vec model—a shallow neural network designed to generate word embeddings based on word collocation—to predict words based on the document identifier and broader lexical context. This information is used to generate document embeddings (i.e., numeric vector representations) for each SDG’s P/I document^15^. Cosine similarity between document embeddings captures discourse proximity or overlap between SDGs in UN reports. These similarities are represented as a weighted network of SDGs, with the weight of each network edge indicating the normalized cosine similarity between UN reports on two SDGs. To identify clusters of similar SDGs, complete-link cluster analysis was conducted on the matrix of cosine similarity scores between SDGs, and the Louvain community-detection algorithm was applied to the network of SDG similarities^39,40^. When switching from a model trained on the UN P/I corpus (Fig. 1) to a model trained on 11.8 GB of US news reports (Figure S4), the average cosine similarity increased to 0.94 (sd = 0.02) but there were no substantive differences in patterns of discursive similarity between SDGs. Results on discursive similarities are also robust to different specifications of doc2vec models and to other NLP methods such as Latent Semantic Analysis (see SI Appendix).

To assess the alignment between overlap in SDG UN reports and integration between SDGs in scientific literature, we collected metadata on all peer-reviewed scientific articles (between 2000 and 2020) concerning at least one of the six SDGs in the three pairs with the strongest cosine similarity scores in UN reports (SDGs 5 and 16, 10 and 17, 14 and 15). These publications were identified using the dimensions.ai SDG classifier^41^, a supervised machine learning algorithm trained on SDG-relevant publications gathered on the basis of a manually curated, extended search string (one for each goal). The resulting publication dataset consisted of 779,901 articles over 21 years (N_2000_ = 12,727 articles, N_2020_ = 67,916, year mean = 37,138, year SD = 21,119), each tagged by the classifier as relevant to one or more of the six SDGs of interest. We first used this dataset to calculate overlap between the six SDGs in scientific literature since 2012. For each pair of SDGs, overlap between their articles is measured with the Jaccard Index, a common measure of alignment between two sets^42^: the proportion of articles classified as relevant to *both* SDGs (set intersection) relative to all articles classified as relevant to *either* SDGs (set union). From the publication dataset we also extracted 21 yearly co-authorship networks of scientists publishing on SDGs in each of the three pairs in 2000–2020. This produced a total of 63 networks (21 years × 3 SDG pairs), with 2210 to 51,270 authors and 1887 to 316,769 collaborative ties. Authors were assigned one of three tags indicating which SDG in each pair their publications contributed to in that year (SDG A, SDG B, SDG A + B). Three measures of integration in SDG scholarship are presented: (1) the Jaccard Index indicates the proportion of authors contributing to both SDGs (SDG A + B) relative to all authors contributing to either SDG in the pair (SDG A, SDG B, or SDG A + B); (2) the average SDG entropy in Louvain communities of the coauthorship network^40,43^ captures the extent to which authors contributing to different SDGs cluster in the same community of collaborating scientists; (3) the assortativity coefficient of the coauthorship network by SDG tag^44^ measures the degree to which collaborations occur between authors with the same SDG tag, with 0 (1) indicating maximum (minimum) collaborative mix between authors with different SDG tags.

## Results

### Extracting key contents from UN SDG Progress and Information (P/I) reports

Several key words and phrases extracted from the reports via TextRank offered initial insights into discursive overlaps and potential interdependencies between SDGs. Patent examples include “biodiversity”, shared by SDGs 14-Aquatic and 15-Terrestrial; “gender”, which appears in SDGs 5-Gender and 4-Education; “right” (as in “equal rights”), common to SDGs 5-Gender, 16-Peace and 10-Inequality; and “energy” in SDGs 7-Energy and 13-Climate. Less expected examples include the key phrase “development assistance,” shared between 2-Hunger, 3-Health, 10-Inequality and 17-Partnerships. Such overlap reveals that UN discourse on the SDGs in this group emphasizes the delivery of aid to developing countries—consistent with theoretical models which classify good health and nutrition as basic human needs—with international partnerships (SDG 17) working towards reducing global inequalities in the achievement of these goals^18^. A more detailed list and visualization of the UN report key words by SDG is provided in the SI Appendix. Many of these words are integral to several SDGs (e.g., “development”, “global”, “sustainable”), with over a quarter of them (559 of the 1969 unique TextRank key words and phrases) being shared by more than one SDG. The relatively high number of shared terms means that no single lexical overlap can adequately summarize the complex network of interdependencies between SDGs based on similarities in UN policy discourse. As an example, while the aforementioned “development assistance” phrase is shared by SDGs 2, 3, 10 and 17, it also partially overlaps with the term “social development”, which is common in reports on an entirely different cluster of SDGs.

### SDG interdependency networks from discursive similarities in UN P/I reports

We represent SDG interdependencies as a network of discursive similarities or overlaps between UN P/I reports about each SDG. Overlap is measured as normalized cosine similarity between doc2vec embeddings of the reports (Fig. 1). Cosine similarity between the SDGs ranged from 0.62 to 0.96 (mean = 0.82, SD = 0.06). These high levels of discursive similarity suggest a strong overall degree of interdependence and potential integration between SDGs. Yet they also capture the recurrence of similar expressions and linguistic syntax in P/I reports, reflecting the highly structured and standardized nature of these documents. SDG 17-Partnerships is the most central goal in the similarity network, with respect to both weighted degree and eigenvector centrality. This indicates that SDG 17 is both characterized by greater discursive overlap with other SDGs and connected to SDGs which in turn are heavily interdependent on each other. This result is consistent with the main purpose of SDG 17: the mobilization of resources and partnerships to address other SDGs. While cosine similarities between 17-Partnerships and other SDGs tend to remain steadily high (~ 0.87), an important exception is the below-average overlap between 17-Partnerships and 13-Climate (cos = 0.76). Indeed, on average, environmental SDGs (12-Consumption, 13-Climate, 14-Aquatic, 15-Terrestrial) exhibit a lower cosine similarity to SDG 17 (mean = 0.82) compared to all other SDGs (mean = 0.89), signaling that the UN’s discussion of progress towards 17-Partnerships tends to favor social and economic rather than environmental goals.

Both the hierarchical complete-link clustering of SDGs by cosine similarity (Fig. 1A) and community detection in the similarity network (Fig. 1B) reveal a clear divide in UN discourse around the goals: environmental SDGs (SDGs 12 to 15)—which focus on terms such as “earth preconditions”, the “natural environment”, “planet” or “biosphere”—are discursively distinct and separated not only from 17-Partnerships, but from all other goals in the network. This is especially the case for 13-Climate, which is assigned its own dendrogram branch, suggesting it tends to be discussed in entirely different terms to the other sixteen SDGs.

The remaining, predominantly social SDGs are divided into three clusters. First, the most central group includes 7-Energy, 8-Economy, 9-Industry, 10-Inequality, and 17-Partnerships. These 5 SDGs share key phrases such as “national development”, “economic growth”, “broadband”, and “trade”, revealing that they are similarly discussed by the UN in terms of macroeconomic and infrastructural development, and of the international cooperation and assistance that facilitate it. The high proximity between SDGs in this cluster is also in part attributable to their reports’ focus on developing regions in Southeastern Asia and Sub-Saharan Africa. A second cluster containing 6-Sanitation and 11-Settlements concerns the infrastructural development of human settlements and urban areas. The cosine similarity between these two SDGs (cos = 0.81) is not especially strong when compared with other pairings. However, shared key words indicate that the UN discusses water resource management regarding both SDGs, albeit in slightly different terms (access for SDG 6 vis-à-vis quality for SDG 11).

The third cluster contains 1-Poverty, 2-Hunger, 3-Health, 4-Education, 5-Gender and 16-Peace, which are broadly related to social and economic development. In this group, SDGs 5 and 16 are the pair with the greatest cosine similarity (cos = 0.92), followed by the triplet of SDGs 1, 2 and 3 (cos = 0.89). Examining overlaps in key phrases, major points of interdependence between 5-Gender and 16-Peace in UN discourse specifically concern issues of sex trafficking and sexual violence, particularly at the hands of intimate partners and during childhood. On the other hand, the UN’s discursive overlap on 1-Poverty, 2-Hunger, and 3-Health is explicit in its focus on financial hardship, poverty, and lack of work. Reports emphasize how these factors can cyclically lead to poor diet and health, which in turn perpetuate inability to work and extreme poverty. Between the two sub-clusters of SDGs 5 and 16, and SDGs 1, 2 and 3, 4-Education is placed almost equidistant, because of its relationship with both 5-Gender (albeit not in the domain of sexual violence) and 1-Poverty. Finally, despite being assigned to a separate dendrogram branch (the first social cluster described above), 8-Economy is also characterized by above-average overlap scores with the triplet of 1-Poverty, 2-Hunger and 3-Health (cos = 0.88–0.91).

### Scientific integration and collaboration for the most strongly interconnected SDG pairs

Different NLP-based similarity and clustering analyses (see SI Appendix) converge in identifying three pairs of SDGs as the most strongly interconnected in UN discourse: 5-Gender and 16-Peace; 10-Inequality and 17-Partnerships; 14-Aquatic and 15-Terrestrial. Such overlaps may both reflect and further promote integration and collaboration in scientific research related to the six SDGs in these three pairs. To examine this issue, we downloaded metadata on all scientific articles published between 2000 and 2020 and tagged as relevant to the six SDGs by the dimensions.ai SDG classifier^41^. Since an article may be tagged by the classifier as pertinent to multiple SDGs simultaneously, a first index of scientific integration between two SDGs is the degree of overlap (Jaccard Index) between articles that are classified as relevant to each.

**Figure 2:**
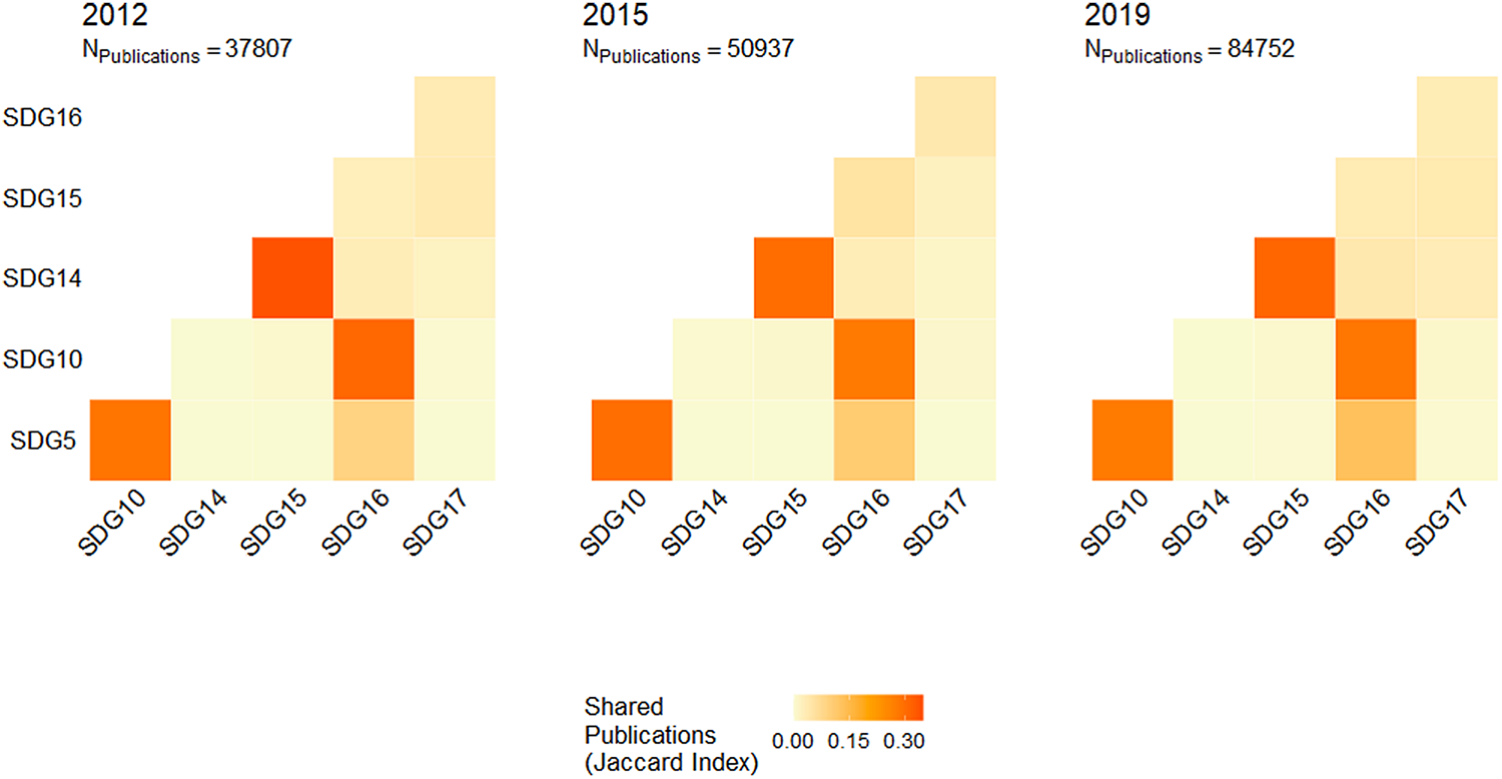
Overlap (Jaccard Index) between scientific articles classified as relevant to SDGs 5-Gender, 10-Inequality, 14-Aquatic, 15-Terrestrial, 16-Peace, and 17-Partnerships since 2012 (the year SDGs were first proposed at the Rio + 20 conference).

A visualization of this index over the years (Fig. 2) shows that integration in SDG-related scholarship does not necessarily align with the UN’s view of SDG interdependencies. The broad shared topics of sustainable use of environmental resources and biodiversity produce great scientific overlap between 14-Aquatic and 15-Terrestrial (0.30 ≤ JI ≤ 0.34, mean = 0.31, sd = 0.02), consistent with UN discourse. However, there is lower scientific overlap between 5-Gender and 16-Peace (0.09 < JI < 0.13, mean = 0.12, sd = 0.01), the most strongly connected pair in UN reports. Furthermore, while far apart in terms of UN discursive affinities, 5-Gender and 10-Inequality are characterized by a substantial degree of intersection in scientific publications (0.27 ≤ JI ≤ 0.30, mean = 0.28, sd = 0.01), likely reflecting their shared focus on reducing inequalities. Similarly, 10-Inequality and 16-Peace are relatively distant in UN discourse but characterized by consistent proximity in scientific literature (0.28 < JI < 0.31, mean = 0.29, sd = 0.01). By contrast, the low scientific overlap between 10-Inequality and 17-Partnerships (0.00 ≤ JI ≤ 0.02, mean = 0.01, sd = 0.00)—despite related UN reports’ shared interest in reducing global inequalities via international cooperation, partnerships, and assistance—suggests that scientific research may be focusing on different types of inequality than those emphasized in international policy discourse.

**Figure 3:**
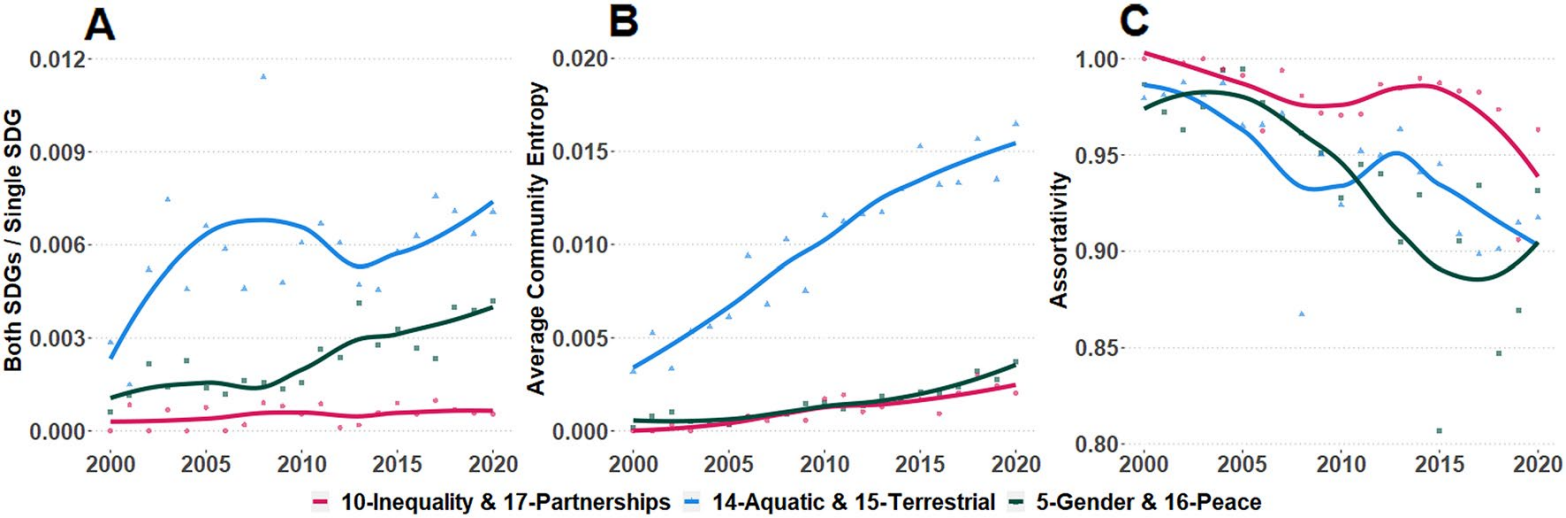
Integration and collaboration between scientists conducting SDG-related research in the three SDGs pairs with strongest interconnection in UN discourse (2000–2020). **(A)** Proportion of scientists working on both SDGs relative to those working on either SDG. **(B)** Entropy of Louvain communities by scientist SDG designation in coauthorship networks. **(C)** Assortativity index by scientist SDG designation in coauthorship networks. Locally estimated scatterplot smoothing (LOESS) is applied to fit a polynomial moving average to the data.

For each of the three SDG pairs of interest, we also constructed yearly co-authorship networks in 2000–2020 (see Figs. S8–S10). In the networks for each pair, authors are designated as working on either or both SDGs each year (based on dimensions.ai SDG classification of their publications). This designation is used to extract measures of collaboration among scientists who conduct research related to different SDGs over the last two decades (Fig. 3). In all three pairs, the overall trend is one of gradually greater integration and collaboration between researchers working on different SDGs. Of the three presented measures of scientific integration, the most stable is the proportion of authors conducting research on both SDGs in the pair (Fig. 3A). This proportion is generally low in all three pairs, suggesting that most authors specialize in expertise that is relevant to a single SDG. However, the measure increased substantially for two pairs (5-Gender and 16-Peace, 14-Aquatic and 15-Terrestrial), particularly in the past decade. Average community entropy, an index of SDG tag diversity in communities of collaborating authors, also grew over time (especially for 14-Aquatic and 15-Terrestrial), indicating that scientific teams increasingly gather authors who work on different SDGs in each pair (Fig. 3B). Finally, the assortativity coefficient decreased considerably over time in all three pairs, demonstrating growing collaboration between authors who contribute to different SDGs over the past 20 years (Fig. 3C).

## Discussion

Since Rio + 20, scholars have advanced a variety of theoretical and empirically grounded models describing interdependencies and clusters between SDGs. While most recent approaches have identified SDG synergies and trade-offs by analyzing indicators of sustainable development in different domains, this study goes back to the source of official, internationally agreed definitions of sustainable development: it maps the SDG interdependencies emerging from discursive similarities in the way goals are discussed in the international policy language of official UN reports. Crucially, these reports both describe current progress and propose future targets: thus, they reflect both features of actual development policies enacted by national governments, and priorities for sustainable development advanced by the UN.

We find moderate-to-high levels of discursive overlap between all SDGs, consistent with the fundamental indivisibility and coherence which, by design, characterize the goals. SDG 17-Partnerships is the most central goal in our interdependency network, which reflects its centrality to the UN’s push for coordinated global action towards all other goals. On the other hand, the goals related to environmental sustainability and ecology—particularly 13-Climate—are in marginal positions in the network and separate from all other goals in cluster analyses. This discursive divide could derive from the relative novelty of the UN environmental agenda compared to the social and economic UN Millennium Development Goals^45^, or perhaps from the higher number of internal trade-offs between sub-targets of the environmental goals compared to other SDGs^28^. The divide could also signal isolation and siloing of climate policy from other sustainability policy domains^3^. Moreover, it could reveal greater difficulty in reaching international policy consensus around environmental rather than social and economic actions, with SDG 13-Climate and other environmental goals being those on which global progress is currently most off-track^3,10,46,47^. The discursive insularity of 13-Climate in our findings, in particular, is in stark contrast with the wide and strong interdependencies, found in previous literature, between climate action and the achievability of most other SDGs^2,3,22^, and raises questions about global capacity to integrate environmental policies with health and economic recovery efforts after COVID-19, a central priority according to recent one-health research^48^.

Our findings are in line with some of the SDG interactions observed in previous research but run counter to others. For example, our results confirm Le Blanc’s observed interaction between 10-Inequality and 16-Peace via “eliminating discrimination”, “developing country participation in global governance” and “political inclusion”^6^. However, in contrast with analyses of shared targets, we also detect a strong relationship between 3-Health and 8-Economy, suggesting a potential mismatch between SDG target overlaps and the SDG interlinkages emerging from progress reports. Additional consistencies with prior research include the centrality of 3-Health^25^ in SDG networks, and the clustering of 1-Poverty, 8-Economy, 11-Settlements, and 17-Partnerships^26^. We also replicated the strong relationship observed between 17-Partnerships and 10-Inequality^11^, which share a focus on international development and inequality. On the other hand, our results differ from SDG networks described in research on specific regions or on developing nations, wherein 1-Poverty, 15-Terrestrial, and 16-Peace take on more central roles^24^.

Comparing SDG interdependencies in international policy language vis-à-vis global science, we find that scientists do not always prioritize the same areas of SDG integration highlighted in UN discourse. Since 2000, scientific collaboration and integration have generally increased in research related to those SDGs that are closest in UN discourse. However, increases are much greater when the goals are focused on narrower objectives (14-Aquatic and 15-Terrestrial). On the other hand, in certain cases scientists tend to see overlaps between SDGs that show little proximity in UN discourse (e.g., 5-Gender and 10-Inequality), while certain SDG interdependencies in UN discourse seem to be overlooked in the scientific literature (e.g., 10-Inequality and 17-Partnerships). While this article does not aim to establish or quantify causal links between UN language and scientific research, a relationship of mutual causality could be hypothesized between the two, with each domain influencing the other over time. The existence of both areas of consistency and misalignment between UN policy discourse and global scientific language suggests important questions and avenues for future research about this causal relationship.

Three clusters of SDGs with strong similarities in UN language, identified by our analysis, have especially important implications for global policies and cooperation after the COVID-19 pandemic. First, *5-Gender and 16-Peace* are the two SDGs with the closest connection in UN reports—confirming a shared focus on “end[ing] discrimination” and supporting “women in leadership roles”^6^. UN discourse derives this link from discussions of gender-based violence and other joint impediments to the two SDGs. However, scientific literature often emphasizes the positive connections between them: empowering women and facilitating their participation in institutions (SDG 5) can positively support the achievement of 16-Peace^49^; conversely, advances towards more inclusive and stronger institutions (SDG 16) can support 5-Gender by improving women’s access to healthcare and female leadership^50,51^. Expanding UN discourse to value the positive synergies observed by researchers between SDGs 5 and 16 will be particularly important in global efforts for post-COVID recovery, given the greater impact that the pandemic is having on women^52–55^.

Second, UN documents reveal a strong interdependence between SDGs *10-Inequality and 17-Partnerships.* This discursive similarity is mostly driven by language around international inequality and cooperation. In contrast, scientific literature related to SDGs 10 and 17 focuses primarily on intra-national dimensions of inequality and their links with themes of central importance to national publics, such as political polarization, the rise of populism, and economic growth^56–58^. Issues of international inequality may become more salient in future scientific research as they gain more prominence following the COVID-19 pandemic, especially with respect to global availability of vaccines, access to medical advances, and instances of healthcare weaponization^59,60^.

A third cluster of highly interconnected SDGs in UN language comprises *3-Health and 1-Poverty, 2-Hunger, and 8-Economy*. The link between 3-Health and SDGs 1 and 2 is consistent with the scientific consensus that health, nutrition, and access to economic resources are strongly associated^61–63^. The interdependence between 3-Health and 8-Economy in UN reports is robust but less strong, a trend consistent with the mixed findings in scientific literature about the causal relationship between economic status and health for both individuals and countries^64–67^. The synergies and trade-offs between 3-Health and economy-related SDGs 1, 2 and 8, which emerge from our analysis of UN documents, are becoming increasingly salient in the wake of COVID-19 as countries strive to protect population health while stimulating economic recovery.

## Conclusions and future directions

The application of network analysis to policy-related texts has recently been the subject of significant research. However, in contrast to the approach proposed in this study, existing methods require preliminary human annotation of documents^68^, which may be impractical, too costly, or affected by subjective biases of the analysts. The combination of NLP methods and network analysis presented here proved effective to map relationships among SDGs as they emerge empirically from reports by policymakers and scientists; to measure interdependencies between SDGs in official policy documents; and to detect points of convergence and misalignment between international policy discourse and scientific research. These methods provide a novel lens and toolkit in the ongoing effort to quantify SDG interlinkages, identify coherent clusters of integrated SDGs, and monitor SDG progress and interactions over time^3,10,47^. Advances in these methods, such as aspect-based sentiment analysis^69^, could even provide a means of automatically quantifying synergies and trade-offs and contribute to on-going efforts to develop longitudinal measures of SDG progress. The computational techniques we adopted will become particularly important as the number of policy reports and scientific publications about sustainable development continues to grow, exceeding human capacity to exhaustively inspect and annotate this literature, and requiring tools to complement human reading, extract quantitative measures from text, and automate the detection of new insights.

As mentioned in our discussion, important differences in the distribution of SDG interactions arise when focusing on specific regions or points in time^24,47,70^ An extended effort to monitor discursive interactions over time and at the national level would provide valuable insights into spatial and temporal variation, as well as the co-evolution and causal relationships between policy and scientific discourse—both globally and nationally. Such an effort would also facilitate real-time analyses of discursive convergence and divergence in reports from national governments and agencies.

Finally, the methods proposed in this article can also help to identify cross-cutting themes or “zipper concepts” which bridge different SDGs and may be addressed by scientists or policymakers to co-advance multiple goals simultaneously^3^. Steering policy or scientific discourse towards these concepts would foster not just discursive integration between SDGs, but also real synergies in efforts to address different sustainable development domains, improving policy coherence in specific sustainability areas. The development of zipper concepts appears particularly urgent to bridge the discursive gap between the environmental SDGs and the rest of the 2030 Agenda, and would contribute to SDG 17-Partnerships with its aim to foster coherence and cooperation between sustainable development policies. Existing literature on SDG interactions with a focus on 13-Climate and other environmental SDGs already offers important suggestions for the identification of such concepts, such as the positive interactions between targets 2.4, 13.1, and 15.1 described by Pham-Truffert and colleagues^3,11,21^. Environmental priorities and their interconnections with human health and wellbeing, life on land and life underwater are becoming more central to global debates as countries recover from the COVID-19 crisis. This shift could be the perfect opportunity to explore and prioritize novel areas of synergy while understanding trade-offs between SDGs. Identifying these novel areas of intersection between SDGs will be crucial to inform adjustments in global policies for sustainability in the post-COVID-19 world.

## Supplementary Information

Supplementary Information.
