# Supplementary material for: Natural language processing and network analysis provide novel insights on policy and scientific discourse around Sustainable Development Goals
Source: Sci Rep. 2021 Nov 17;11:22427. doi: 10.1038/s41598-021-01801-6 (PMC8599416; doi:10.1038/s41598-021-01801-6)
Supplement: Supplementary file 1 — Supplementary Information. [file 41598_2021_1801_MOESM1_ESM.pdf]

Supplementary Information for

Natural language processing and network analysis provide novel insights on policy and scientific discourse around Sustainable Development Goals

Thomas Bryan Smith*[a], Raffaele Vacca[b], Luca Mantegazza[c], Ilaria Capua[c]

[a] Bureau of Economic and Business Research, University of Florida, Gainesville, USA
[b] Department of Sociology and Criminology & Law, University of Florida, Gainesville, USA
[c] One Health Center of Excellence, IFAS, University of Florida, Gainesville, USA
* Corresponding author.

Email: thosmi@ufl.edu

Supplementary Text

Text preprocessing and Part-of-Speech tagging

UDPipe's treebank dependency parser was applied to the UN P/I reports[1–5] to generate the Part-Of-Speech (POS) annotations used in all subsequent analyses. UDPipe is a multilingual Natural Language Processing (NLP) suite found in the R package repository CRAN and on GitHub, including pipelines for tokenization, POS tagging, lemmatization, and dependency parsing[6]. The extracted lemmatization and POS tags were applied at different stages of the analysis.

Text preprocessing for TextRank analysis. As has become common in applied NLP[7,8], our analysis focused exclusively on lemmatized nouns and adjectives – forming the topic words or phrases of a corpus - when extracting SDG keywords via TextRank. Specifically, TextRank's 5-word moving window was applied to the corpus in such a way that only nouns and adjectives are assigned a node in the resulting graph, with connections only emerging between these nouns and adjectives if they appear within 5 words of each other and are related by POS tags (e.g., "The availability of water takes priority, rather than short term macroeconomic growth" would connect "availability" with "water" and "priority", but does not connect "priority" with "macroeconomic" or "growth"). Stopwords were omitted from this analysis after being detected with the *stopwords* function of the R TextMining (TM) package[9]. After qualitatively examining the UN's SDG Progress and Information (P/I) text, we added the following words to the standard list of stopwords provided by the TM package: average, real, many, level, data, total.

Text preprocessing for doc2vec analysis. A slightly different preprocessing pipeline was adopted for this part of the analysis. The self-supervised continuous bag-of-words (CBOW) Word2Vec model, which underlies the doc2vec model, moves beyond simply placing words within a network, and learns to predict words given recurring contexts (e.g., "development" may be predicted from the context "sustainable … goals")[10,11]. In this analysis, focusing exclusively on nouns and adjectives would remove critical information from the word contexts from which Word2Vec learns. Thus, we applied a more nuanced preprocessing pipeline. For each text, we identified, extracted, and concatenated all lemmatized words with POS tags from the Penn Treebank project [12], which distinguishes adverbs (RB tag), determiners (DT tag), verbs in different modes (VB and related tags), and numerous other parts of speech in addition to nouns and adjectives. POS tags were appended to each word (e.g. "goal" → "goal//NN"), ensuring that our Word2Vec model only recognized words as synonyms when they occupied the same POS (e.g. "chosen" as the past participle of choose would be "chosen//VBN", whereas "chosen" as an adjective would be "chosen//JJ"). This more precise POS procedure allowed our model to generate more accurate document embeddings, better navigating linguistic nuances by recognizing that identical words take on different meanings dependent upon their part-of-speech position.

Salient contents in SDG P/I reports: TextRank analysis

We applied the TextRank algorithm to succinctly describe contents in the Progress and Information (P/I) document pertaining to each SDG. TextRank is a network-based model for identifying key words and phrases in a text document[13]. The pre-trained UDPipe treebank parser allowed us to identify all nouns and adjectives, the parts of speech most likely to contain topic words. The TextRank algorithm generates an 8-word collocation network (graph) where each node is a word and each link represents the co-occurrence between two words within an 8-word window. Words are then ranked based on Google's PageRank centrality score in this collocation network[14].

TextRank identifies single key words, but it does not directly detect key *phrases*. Being built on a graph representation of text, it does not directly handle n-grams (i.e., contiguous sequences of words): as in all centrality measures, PageRank centrality is calculated for each graph node (i.e., one word), not for sequences of nodes. Thus, we obtained key phrases by (1)

identifying frequently occurring n-grams of 2 to 8 words, and (2) obtaining phrase-level PageRank centrality as the average PageRank score of the component words of each n-gram.

Results from this descriptive analysis are presented in Figure S1. To ensure cross-SDG comparability, PageRank scores were normalized by SDG. The 17 SDG P/I documents are heavily overlapping in their language use. Indeed, 306 (20%) of the 1,599 unique key words and phrases identified across all 17 P/I text were shared by one or more SDGs. Most scored low or exceptionally high on PageRank, as opposed to the low-to-middle PageRank scores of SDG-specific keywords (Fig. S1 B). Figure S2 depicts results from an analysis mapping SDG overlaps based on *exactly* matching TextRank keywords. Jaccard index was used to represent the proportion of words and phrases shared by a pair of SDGs over their complete set of key words and phrases[15]. Using complete-link clustering on the resulting matrix of Jaccard similarities, we extracted the following three clusters of SDGs: (1) 10-Inequality, 2-Hunger, 6-Sanitation, 16-Peace, 5-Gender; (2) 8-Economy, 1-Poverty, 4-Education, 17-Partnerships, 3-Health; (3) 9-Industry, 7-Energy, 11-Settlements, 12-Consumption, 14-Aquatic, 13-Climate, 15-Terrestrial. Unlike the topic model-based replications presented below, these clusters partially depart from the doc2vec results reported in the main text. However, they do confirm a few strong interconnections reported in the main text analysis, such as those between 5-Gender and 16-Peace, 8-Economy and -Health, or 14-Aquatic and 15-Terrestrial. The difference in results between the analysis of TextRank keyword overlaps, on the one hand, and topic models and doc2vec analyses, on the other, are expected given that topic models and doc2vec are designed to recognize synonyms in addition to exact word matches, as discussed in the main text. The grouping of 5-Gender and 16-Peace indicates that they match very closely in their key word/phrase use, suggesting substantially recurring language therein. In contrast, the increased distance between 17-Partnerships and 10-Inequality compared to the main text analyses indicates that this pair of SDGs do *not* use identical language, but rather similar language (or synonyms) in similar word contexts, determining their much stronger relationships in our topic models.

Similarities between SDGs in UN discourse: robustness checks using alternative model specifications and NLP methods

Doc2vec models with different numbers of hidden layers. Considering the relatively small size of our documents, we performed sensitivity analyses while training our doc2vec models to ensure the stability of reported results. As is convention, the model reported in the main text is specified to include 300 hidden layers (dimensions), with 250 iterations, a 5-word moving window, and negative sampling[16,17]. During training, we iteratively increased the number of hidden layers in the model (10, 50, 100, 150, and 300) until we reached a point where embeddings resulted in stable clusters. As can be seen in Fig. S3, a clear divide between anthropocentric and environmental SDGs emerged regardless of model specification. However, there was variation in the hierarchical clustering of the anthropocentric SDGs. At 300 hidden layers we observe a minor variation in the placement of 4-Education between clusters. We suspect that this SDG’s tendency to float between the pairing of 5-Gender and 16-Peace, and the triplet of 1-Poverty, 2-Hunger, and 3-Health, is indicative of the broad relevance of 4-Education to both issues of gender inequality *and* poverty (and its correlates). Nevertheless, 4-Education is consistently placed within the individual-level anthropocentric SDGs, while 9-Industry, 8-Economy, 17-Partnerships, 10-Inequality, and 7-Energy are all placed in the macro anthropocentric SDG cluster – all consistent with the model reported in the main text. The three closely connected SDG pairs examined in the main text (5-Gender and 16-Peace, 10-Inequality and 17-Partnerships, 14-Aquatic and 15-Terrestrial) emerge consistently across all model specifications, and so too does the broader distinction between macro and individual-level SDGs (except for the 10 hidden layer model). However, as we reduce the number of hidden layers, the anthropocentric clusters become increasingly unstable, particularly in the placement of 6-Sanitation, 7-Energy, and 11-Settlements with lower numbers of hidden layers. This instability is to be expected given that our documents are a set of policy texts with a greatly overlapping dictionary of words pertaining to sustainability, meaning that more iterations are necessary to sample the scant unique words and contexts emblematic of the differences between the SDGs (see Fig. S1).

Doc2vec models pre-trained on US news report corpus. While the doc2vec model presented in the main text relies on word embeddings trained on the UN P/I report corpus, we also tested the sensitivity of our results to a pre-trained doc2vec model. This model was trained (CBOW with 500 hidden layers, 10-word moving window, and negative sampling) on 11.8GB of online US news reports from numerous sources (Reuters, New York Times, LA Times, etc.). The news reports include 2.3 billion words overall and can be downloaded from the word2vec++ GitHub repository[18]. Results from this analysis, as visualized in Figure S4, are largely consistent with the clusters and interdependencies identified in our main model. For example, 11-Settlements and 6-Sanitation were paired off separately to the rest of the SDGs. Environmental SDGs (12-Consumption, 13-Climate, 14-Aquatic, 15-Terrestrial) were assigned their own cluster. The strongest pairings (10-Inequality & 17-Partnerships; 14-Aquatic & 15-Terrestrial; 5-Gender & 16-Peace) were replicated.

However, there is one notable departure from the results presented in the main text: 1-Poverty and 2-Hunger form a distinct cluster with 10-Inequality and 17-Partnerships, respectively separating from the individual-level anthropocentric SDGs (3-Health, 4-Education, 5-Gender, 16-Peace) and macroeconomic SDGs (7-Energy, 8-Economy, 9-Industry). The reason for this alteration is complex, and contingent upon what the Word2Vec model has learned from the US News corpus. Recall that, during the training process, Word2Vec learns word associations based on the use of words in similar contexts[11]. Given that the alternative model was trained on the US news corpus, it has learned word associations from US news articles. Thus, the synonyms which inform doc2vec's representation of the relationships between the SDGs are now learned *a priori* from sources beyond the UN. Indeed, the reason we chose to report the model trained on the UN P/I corpus in the main article (despite the UN P/I corpus being much smaller than the US news corpus) is that the UN text reflects UN's views, priorities, and observations about SDGs and their interdependencies – *not* the more generalist representation of SDG-related topics and language appearing in US news media. Discrepancies between the two models might result from the UN P/I text providing the doc2vec model with a better 'understanding' of the constituent language. It may also be that, compared to UN text, US News outlets tend to more frequently discuss global inequality in terms of poverty and hunger, rather than access to education, public health, gender, and institutional stability.

Doc2vec models applied separately to P/I reports from different years. As a final validation step for doc2vec, we re-applied our final model to the UN SDG P/I documents disaggregated by year. This disaggregation produced 85 texts: 17 SDGs x 5 years between 2016 and 2020. The goal of this validation step was to test the model's ability to accurately group together all yearly iterations of each SDG. As can be seen in Figure S5, we achieved largely accurate results when applying complete-link clustering to document cosine similarity scores extracted from the doc2vec model presented in the main text. Figure S5 highlights both 5 groups, the same number of SDG clusters discussed in the main text; and 17 groups, corresponding to the 17 SDGs. A few major similarities are visible between the 5 clusters identified in the aggregated and disaggregated analyses. In particular, the stark divide between environmental and anthropocentric SDGs is maintained. The most noteworthy departure in the disaggregated analysis is that (a) 1-Poverty was placed in the same cluster as 10-Inequality (despite the cluster's focus relevant to 6-Sanitation, 7-Energy, 11-Settlements, 17-Partnerships), and (b) 2-Hunger is included in the Environmental cluster alongside 14-Aquatic and 15-Terrestrial.

When dividing the 85 texts into 17 groups (one for each SDG), our model successfully grouped all 5 annual P/I documents for SDGs 1, 3, 4, 6, 11, 13, 14, and 15. Moreover, 12-Consumption was correctly isolated into two distinct groups (2016-2018 and 2019-2020), suggesting a shift in how this SDG was discussed post-2018. Consistent with results from the main analysis, our model struggled to separate P/I documents for two of the strongest pairs discussed in the main text: 5-Gender and 16-Peace, 10-Inequality and 17-Partnerships. It created two combined clusters, gathering the 10 annual documents of each pair. The model, however, was able to correctly distinguish 14-Aquatic and 15-Terrestrial (the third strongest pair in the main

analysis) in two separate clusters. Finally, the 10 documents of 8-Economy and 9-Industry were also gathered in the same cluster, consistent with the strong connection between these two SDGs detected in the main analysis. Ultimately, the model we trained on the P/I text was well equipped to identify groups of texts discussing the same SDG. An additional doc2vec model trained on the US News corpus (described above) and applied to the 85 disaggregated annual P/I documents, identified largely the same 5 and 17 clusters (Figure S6), providing further evidence that our main results are not sensitive to either the text corpus used for model training, or the aggregation of annual P/I documents by SDG.

Latent Semantic Analysis trained on Term Frequency-Inverse Document Frequency. We also tested the robustness of our results to an entirely different NLP method, namely, Latent Semantic Analysis (LSA) based on a weighted document-term matrix of term frequency-inverse document frequency (TF-IDF) (Fig. S7)[19,20]. Following the procedure described by Deerwester et al., we converted the 17 P/I documents into a term-document matrix (TDM) [21]. Each matrix row represents a term within one or more of the 17 documents, and each column is one of the 17 SDG P/I documents. Stopwords (including a manually curated set of words unique to the SDG P/I text, such as “per”, “cent”, “average”, “total”) and words with fewer than 4 occurrences were removed from the TDM. We further subset the TDM to only include words with the specific Penn Treebank POS tags (singular noun, plural noun, singular proper noun, plural proper noun, adjective, comparative adjective, superlative adjective). Term frequencies were then converted to TF-IDF, weighting each term proportionally to its frequency in each document and to the inverse frequency of documents containing the term, so that popular terms carried less weight when singular value decomposition was then applied to the matrix.

There are strong similarities between the doc2vec results presented in the main text and the LSA results. There is again a stark divide between the environmental and anthropocentric SDGs. Like in the doc2vec analysis, the three most strongly connected pairs are 5-Gender and 16-Peace, 10-Inequality and 17-Partnerships, 14-Aquatic and 15-Terrestrial. Like the results of the main analysis, 1-Poverty, 2-Hunger, 3-Health, 4-Education, 5-Gender, and 16-Peace are all relatively proximal, as are the infrastructural SDGs of 6-Sanitation and 11-Settlements. The hierarchical clustering of the LSA results depart from the main results only in pairing 8-Economy with 1-Poverty, rather than with 9-Industry. However, the results presented in Figure 1a clearly show a strong cosine similarity between 1-Poverty, 8-Economy (cos = .91), and 9-Industry (cos = .87). It is simply an instance where the consistent, but marginally weaker ties of 1-Poverty to 2-Hunger, 3-Health, 4-Education, 5-Gender, and 16-Peace ($.84<cos<.88$, mean = .87, sd = .02) outweigh the relationships between 1-Poverty, 8-Economy, and 9-Industry when assigning clusters. This is illustrative of the limitations inherent in conceptualizing SDGs as clearly defined, bounded clusters instead of a network of complex interdependencies.

Coauthorship networks of researchers affiliated with the three pairs of highly connected SDGs

Figures S8 through S10 are visualizations of the 3 networks depicting peer-reviewed publication co-authorship on papers relevant to each of the three pairs of closely related SDGs examined in the main text (5-Gender and 16-Peace, 10-Inequality and 17-Partnerships, 14-Aquatic and 15-Terrestrial). All years (2000-2020) are pooled together for each network. The constituent, yearly networks are described in more detail in the Materials and Methods section of the main article.

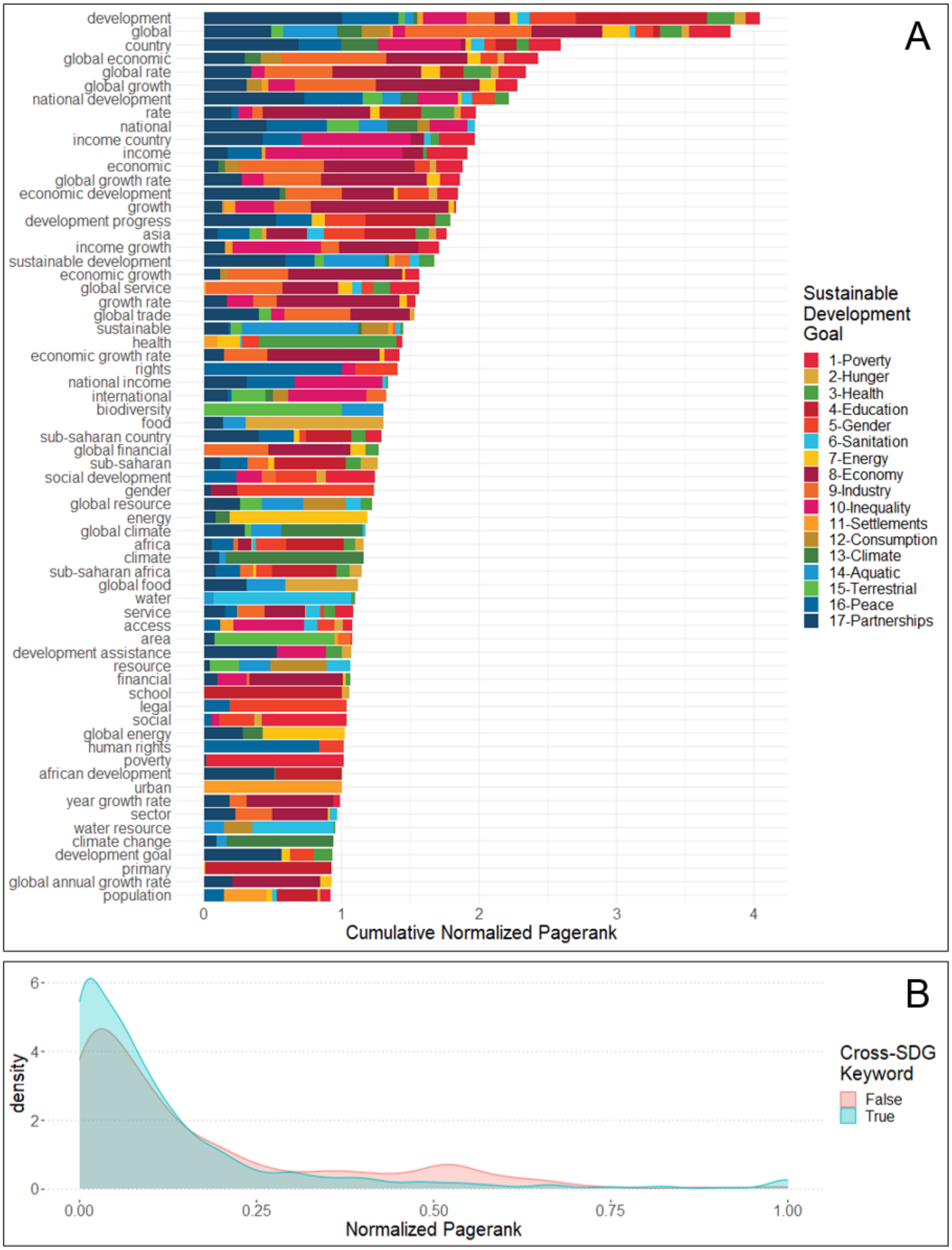

Fig. S1. A: Distribution of SDG TextRank key words and key phrases across SDG. B: Cumulative normalized PageRank centrality.

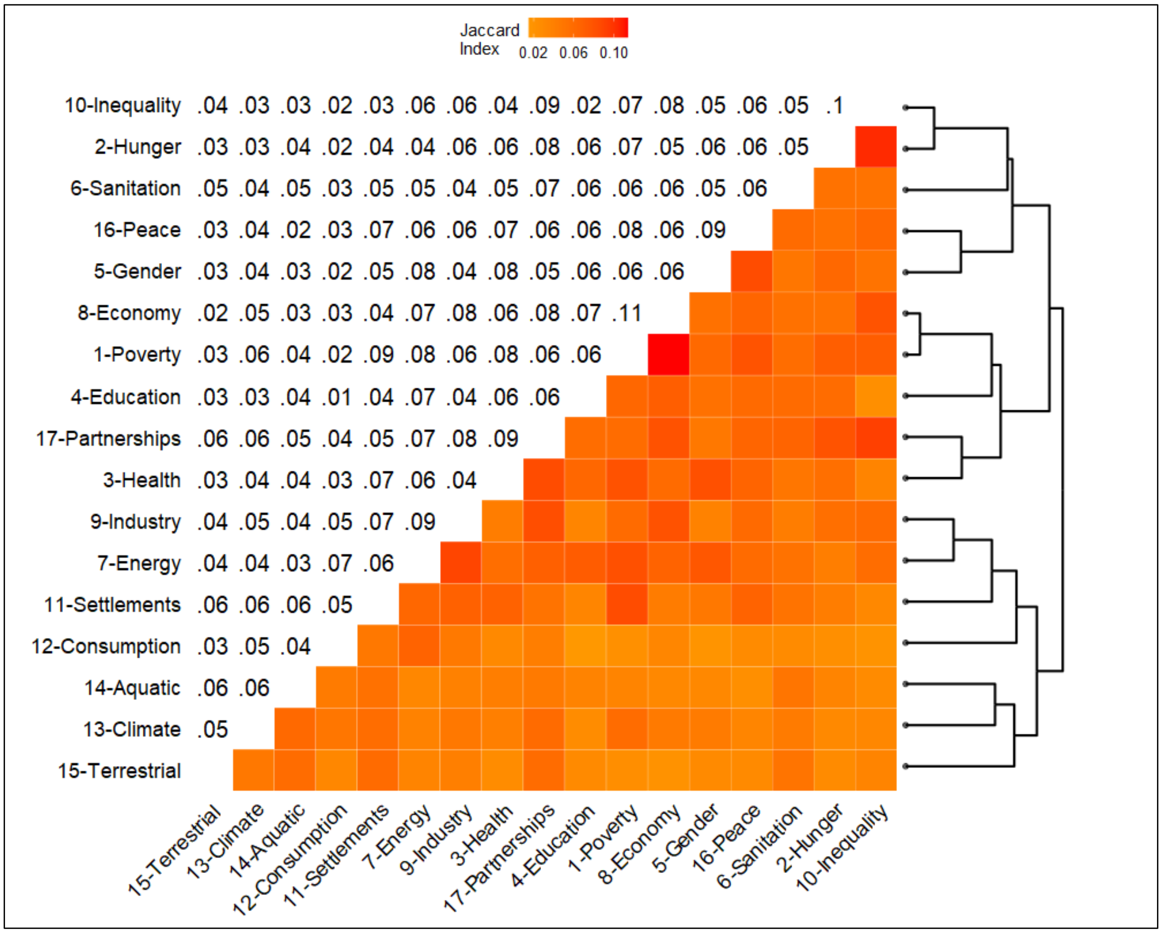

Fig. S2. Jaccard Index quantifying overlap in keyword sets between SDG P/I text

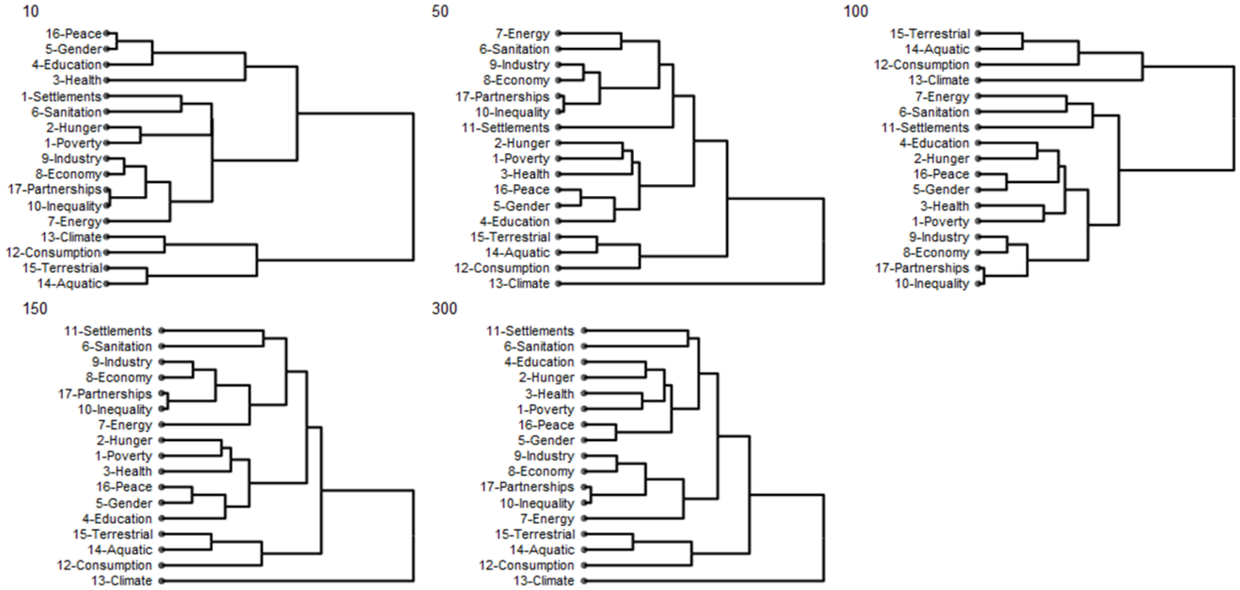

Fig. S3. Example clusters from doc2vec model training by the number of hidden layers

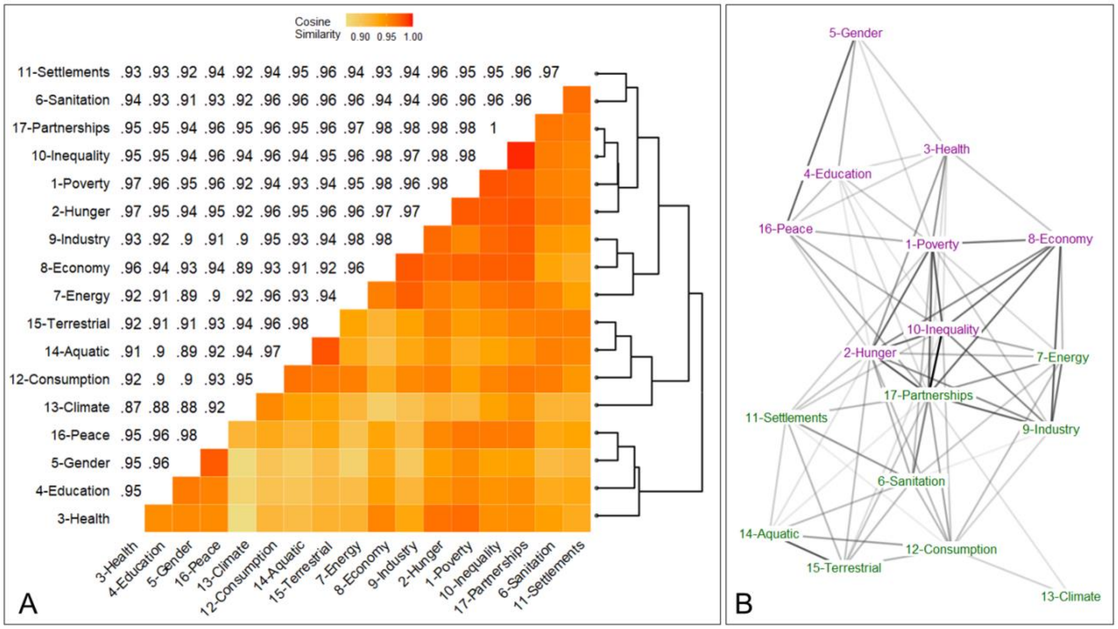

Fig. S4. Cosine similarity matrix reporting cosine coefficients between SDG doc2vec embeddings, model trained on 11.8GB of US News Articles from a variety of sources (Reuters, LA Times, etc.)

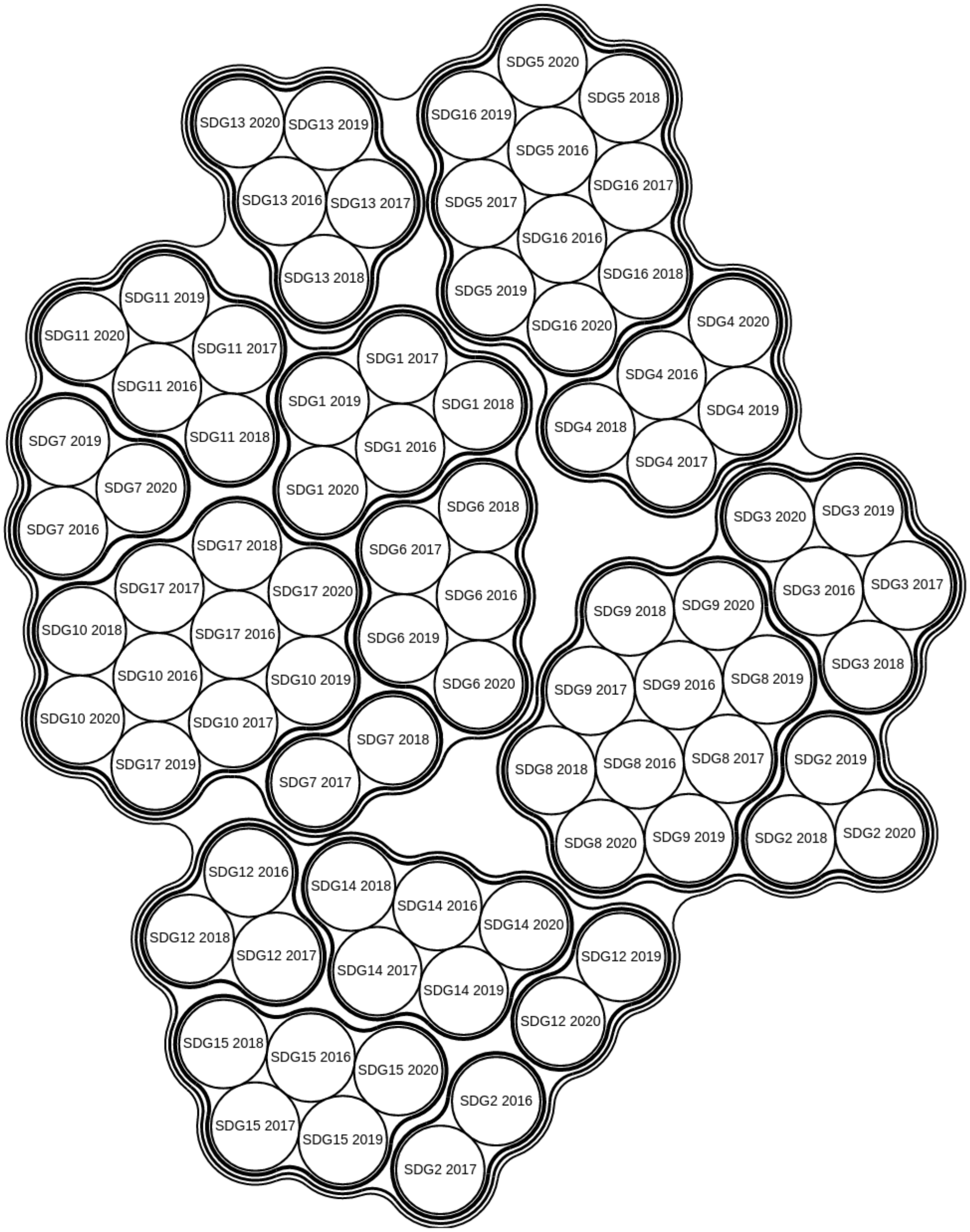

Fig. S5. Circlepack of the SDG P/I documents disaggregated by year as a validation of the doc2vec model presented in the main text.

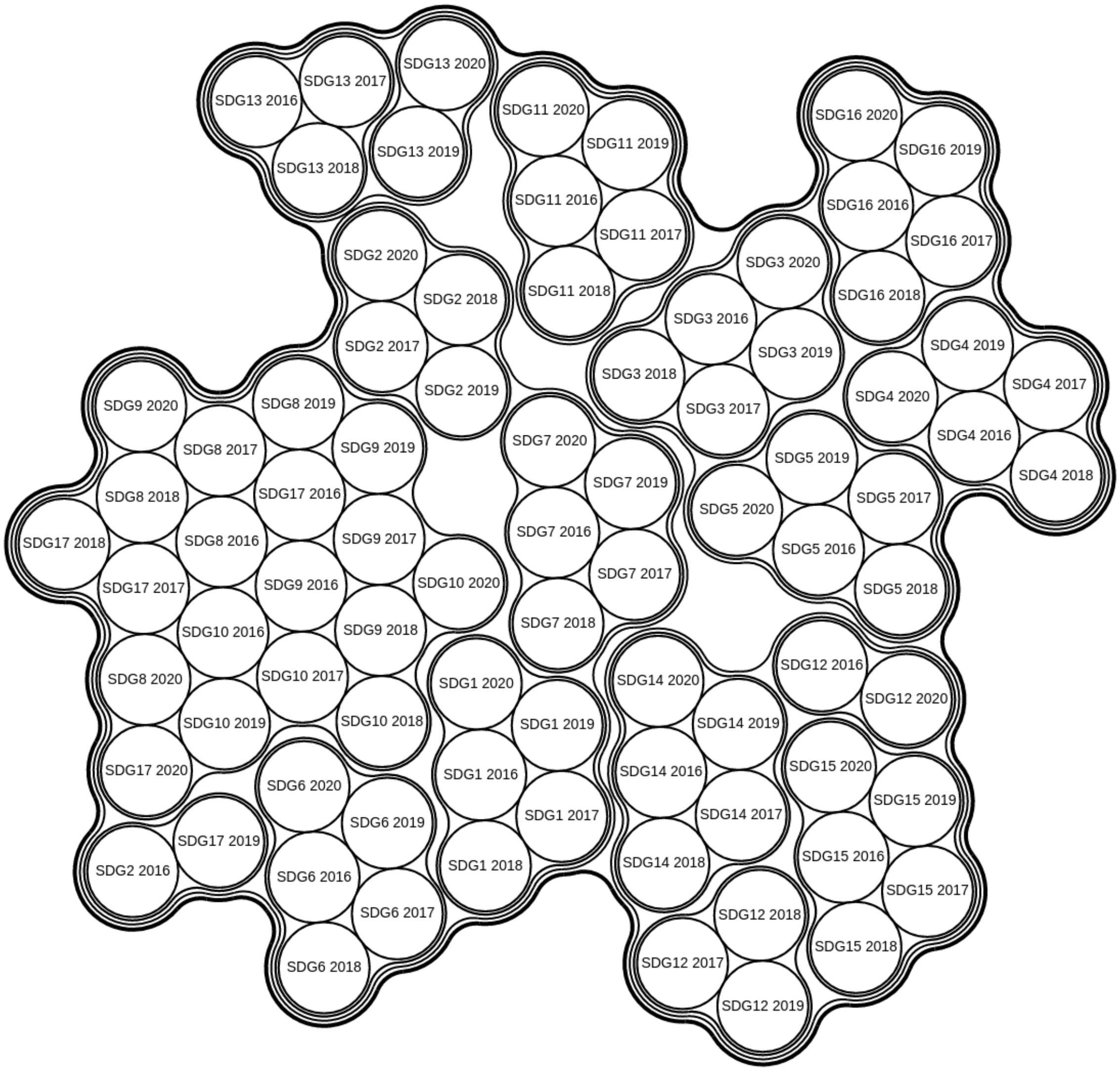

Fig. S6. Circlepack of the SDG P/I corpora disaggregated by year, validation model using the pre-trained US News doc2vec model presented in the supplemental text

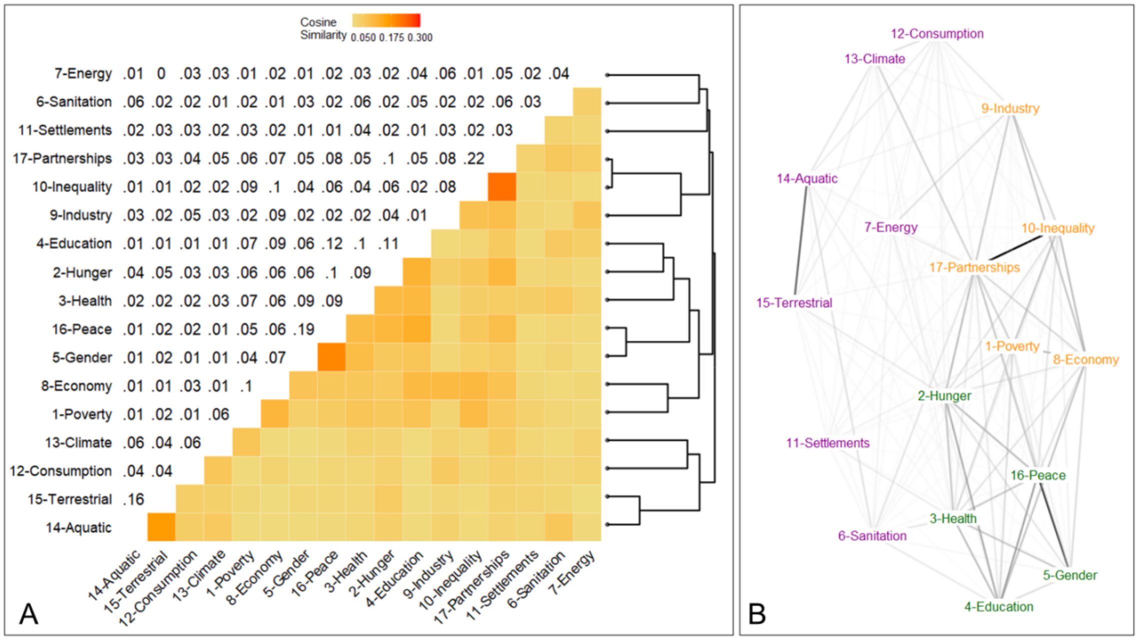

Fig. S7. Cosine similarity between SDG SVD + TF-IDF document embeddings, no network edge threshold

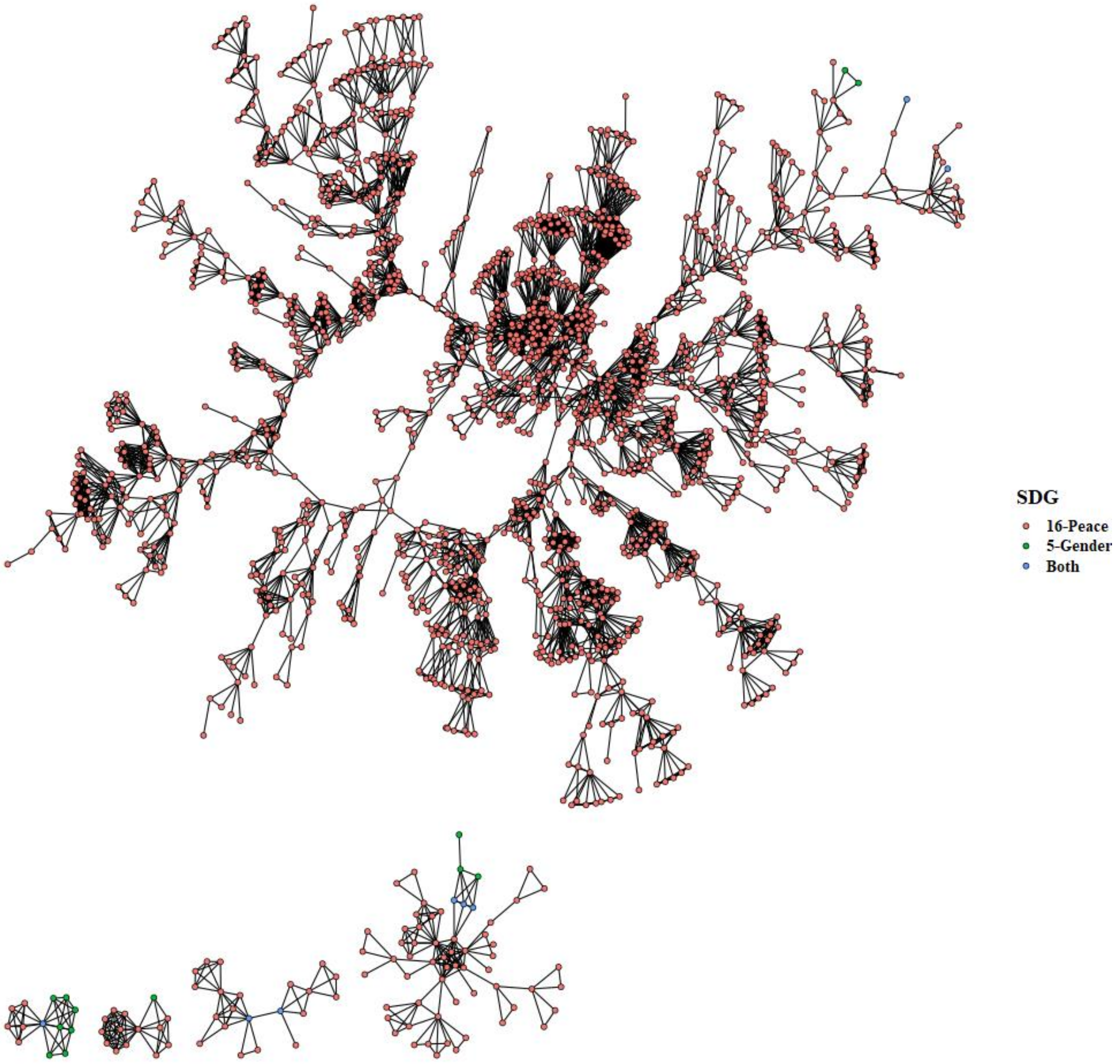

Fig. S8. Largest components (N > 10) in a network mapping collaboration between researchers on publications relevant to SDGs 5 (Gender) and 16 (Peace) (2000 – 2020).

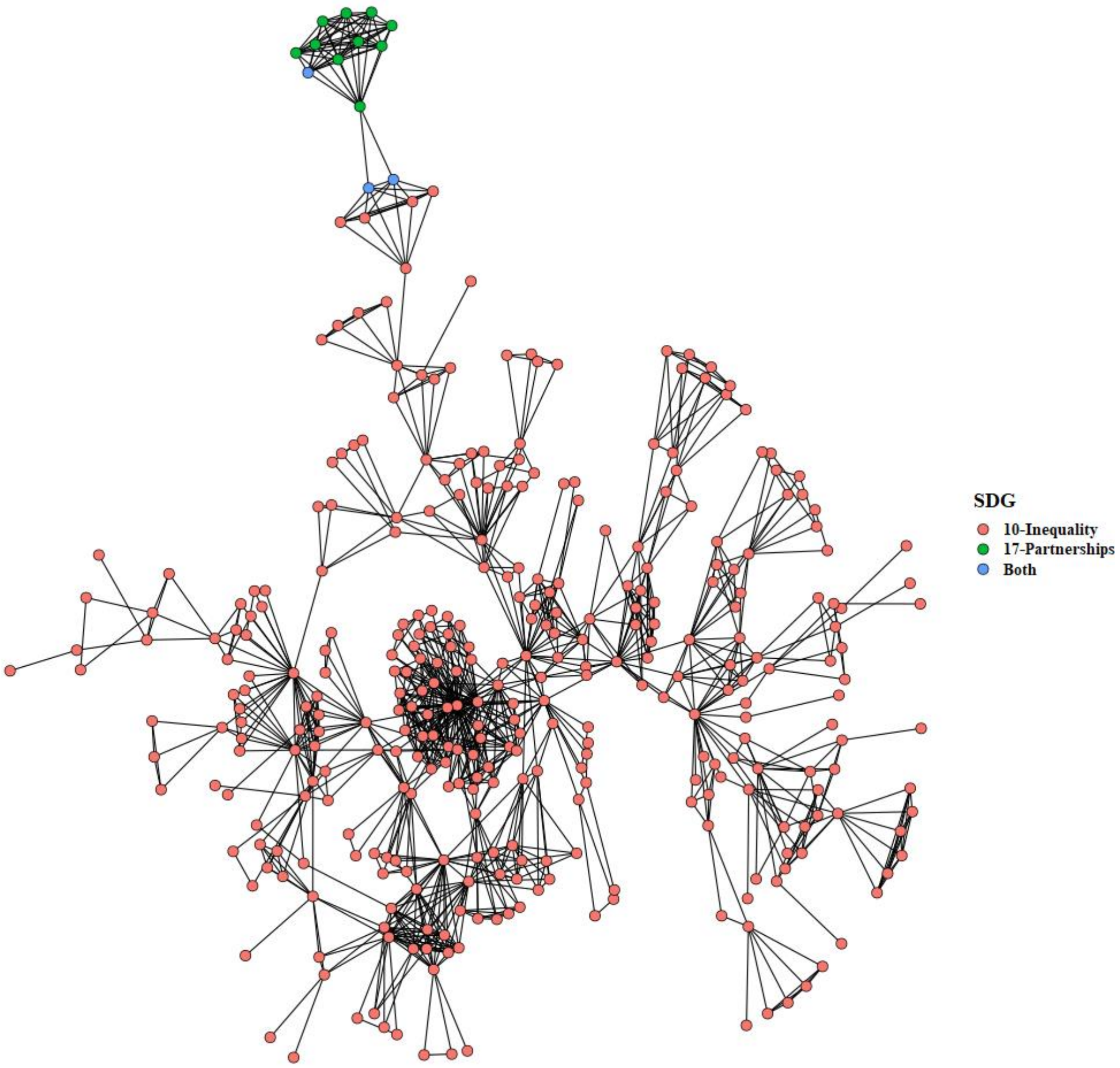

Fig. S9. Largest components (N > 10) in a network mapping collaboration between researchers on publications relevant to SDGs 10 (Inequality) and 17 (Partnerships) (2000 – 2020).

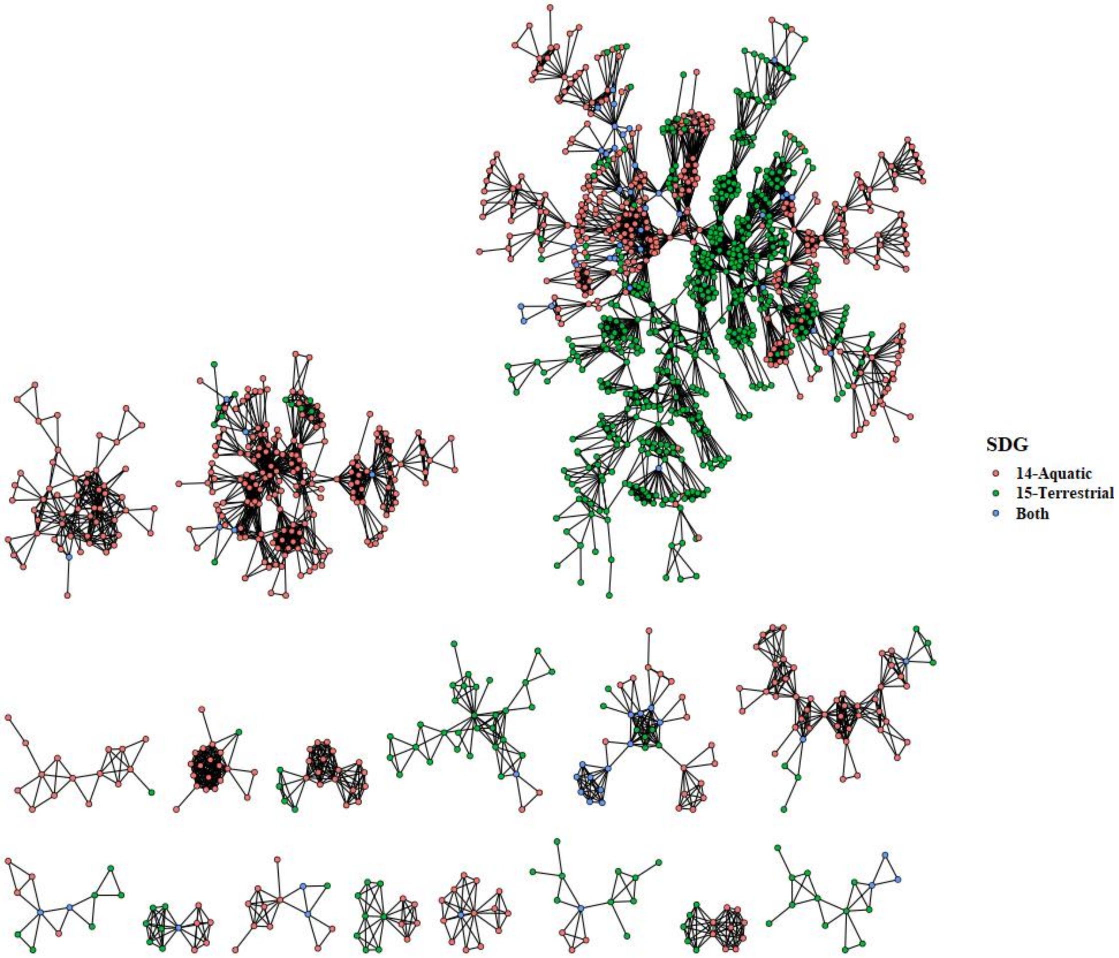

Fig. S10. Largest components (N > 10) in a network mapping collaboration between researchers on publications relevant to SDGs 14 (Aquatic) and 15 (Terrestrial) (2000 – 2020).

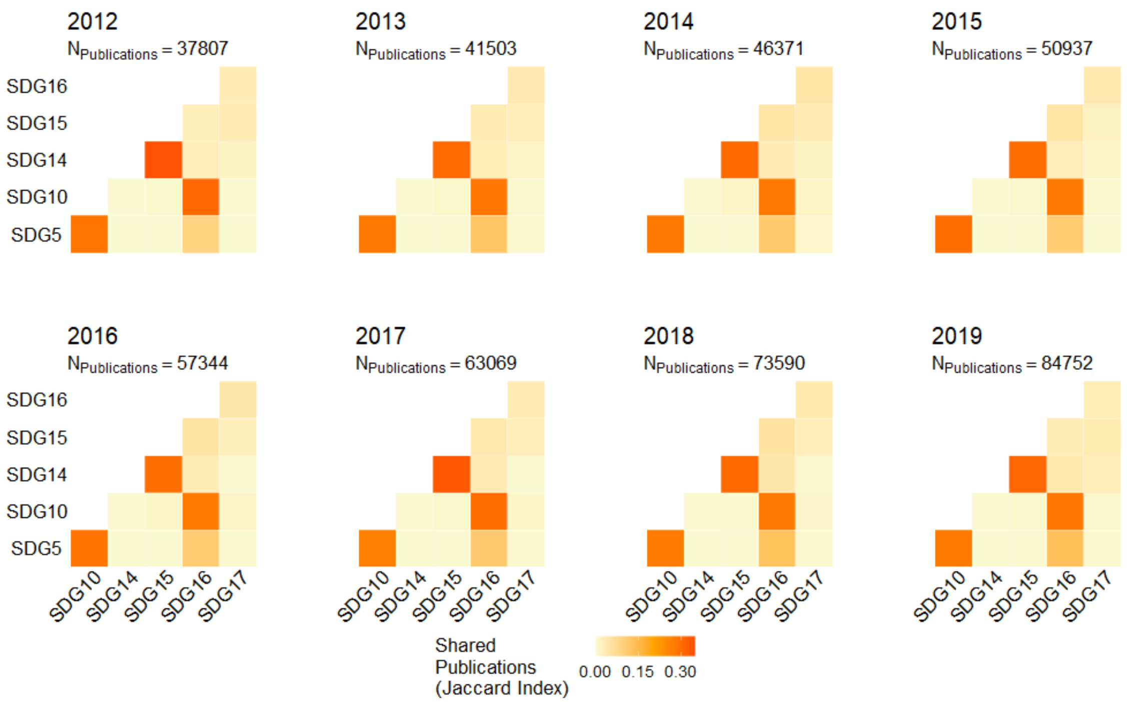

Fig. S11. Overlap (Jaccard Index) between scientific articles classified as relevant to SDGs 5-Gender, 10-Inequality, 14-Aquatic, 15-Terrestrial, 16-Peace, and 17-Partnerships since 2012 (the year SDGs were first proposed at the Rio+20 conference). Complete version of Figure 2, presented in the main text.

SI References

1. ECOSOC. *Progress towards the Sustainable Development Goals - Report of the Secretary-General.* undocs.org/E/2016/75 (2016).
2. ECOSOC. *Progress towards the Sustainable Development Goals - Report of the Secretary-General.* undocs.org/E/2017/66 (2017).
3. ECOSOC. *Progress towards the Sustainable Development Goals - Report of the Secretary-General.* undocs.org/E/2018/64 (2018).
4. ECOSOC. *Special edition: progress towards the Sustainable Development Goals - Report of the Secretary-General.* undocs.org/E/2019/68 (2019).
5. ECOSOC. *Progress towards the Sustainable Development Goals - Report of the Secretary-General.* undocs.org/E/2020/57 (2020).
6. Straka, M. & Straková, J. Tokenizing, POS Tagging, Lemmatizing and Parsing UD 2.0 with UDPipe. in *Proceedings of the CoNLL 2017 Shared Task: Multilingual Parsing from Raw Text to Universal Dependencies* 88–99 (Association for Computational Linguistics, 2017). doi:10.18653/v1/K17-3009.
7. Jing Jiang; Modeling Syntactic Structures of Topics with a Nested HMM-LDA. in *2009 Ninth IEEE International Conference on Data Mining* 824–829 (2009).
8. Darling, W. M., Paul, M. J. & Song, F. Unsupervised Part-of-Speech Tagging in Noisy and Esoteric Domains with a Syntactic-Semantic Bayesian HMM. in *Proceedings of the 13th Conference of the European Chapter of the Association for Computational Linguistics* 1–9 (2012).
9. Feinerer, I. *Introduction to the tm Package: Text Mining in R*. https://cran.r-project.org/web/packages/tm/vignettes/tm.pdf (2020).
10. Mikolov, T., Chen, K., Corrado, G. & Dean, J. Efficient estimation of word representations in vector space. in *1st International Conference on Learning Representations, ICLR 2013 - Workshop Track Proceedings* (International Conference on Learning Representations, ICLR, 2013).
11. Le, Q. V. & Mikolov, T. Distributed Representations of Sentences and Documents. *31st Int. Conf. Mach. Learn. ICML 2014* **4**, 2931–2939 (2014).
12. Santorini, B. *Part-of-Speech Tagging Guidelines for the Penn Treebank Project*. https://repository.upenn.edu/cgi/viewcontent.cgi?article=1603&context=cis_reports (1991).
13. Mihalcea, R. & Tarau, P. *TextRank: Bringing Order into Texts*. https://www.aclweb.org/anthology/W04-3252 (2004).
14. S. Brin, L. Page, The anatomy of a large-scale hypertextual Web search engine. *Computer Networks and ISDN Systems* **30**, 107-117 (1998)
15. Chung, N. C., Miasojedow, B., Startek, M. & Gambin, A. Jaccard/Tanimoto similarity test and estimation methods. *BMC Bioinformatics* **20**, 644 (2019).
16. Rong, X. *word2vec Parameter Learning Explained*. Preprint at http://arxiv.org/abs/1411.2738 (2014).
17. Google. word2vec. https://code.google.com/archive/p/word2vec/ (2016).
18. Fomichev, M. word2vec++. https://github.com/maxoodf/word2vec (2021).
19. Landauer, T. K., Foltz, P. W. & Laham, D. An introduction to latent semantic analysis. *Discourse Process.* **25**, 259–284 (1998).
20. Jones, K. S. A statistical interpretation of term specificity and its application in retrieval. *Journal of Documentation* **28**, 11–21 (1972).
21. Deerwester, S., Dumais, S. T., Furnas, G. W., Landauer, T. K. & Harshman, R. Indexing by latent semantic analysis. *J. Am. Soc. Inf. Sci.* **41**, 391–407 (1990).
